# Natural Killer Cells and Immunotherapy

**DOI:** 10.3390/ijms24108760

**Published:** 2023-05-15

**Authors:** Ji-Yoon Noh, Haiyoung Jung

**Affiliations:** 1 Aging Convergence Research Center, Korea Research Institute of Bioscience and Biotechnology (KRIBB), Yuseong-gu, Daejeon 34141, Republic of Korea; nohj16@kribb.re.kr; 2 Immunotherapy Research Center, Korea Research Institute of Bioscience and Biotechnology (KRIBB), Yuseong-gu, Daejeon 34141, Republic of Korea; 3 Department of Functional Genomics, Korea University of Science and Technology (UST), Yuseong-gu, Daejeon 34113, Republic of Korea

Natural killer (NK) cells are innate immune cells that demonstrate cytolytic activity against tumor cells, virus-infected cells and other physiologically stressed cells, such as senescent cells. The scientific community continues to discover novel biological aspects of NK cells in different organs, tumor microenvironments, and age groups. Recently, cancer immunotherapy has achieved exceptional success. Novel agents and combination therapies that modulate the immune system, including NK cells, provide promising routs for future treatment. 

This Special Issue of the *International Journal of Molecular Sciences*, entitled “Natural Killer Cells and Immunotherapy”, includes eight contributions: five original articles and three reviews that each provide new information on NK cell biology and immunotherapy. 

In their review “The Biological Role and Therapeutic Potential of NK Cells in Hematological and Solid Tumors”, Velichinskii et al. [1] reviewed the role of NK cells in the pathogenesis of hematological and solid tumors and also discussed the current progress in NK cell-based therapy. The authors intensively expounded upon the advantages of NK cells in anticancer immunotherapy and the influence of the tumor microenvironment (TME) on NK cell function. They reported that NK cells have enhanced antitumor potency via innate cytotoxic activity caused by different mechanisms involving the production of cytotoxic granules containing perforin and granzymes, multiple activating receptors, and antibody-dependent cytotoxicity (ADCC). The researchers also demonstrated that many different sources of NK cells, as well as their safe use, can facilitate their application in research and clinical trials. Finally, they explained the current approaches in hematological and solid tumor treatments using NK cell-based immunotherapy. 

In the review entitled “Postoperative Natural Killer Cell Dysfunction: The Prime Suspect in the Case of Metastasis Following Curative Cancer Surgery”, Market et al. [2] introduced NK cells as critical mediators of metastasis formation after curative cancer surgery. The authors introduced the history of cancer surgery and the paradox of surgical resection, which they linked to increased metastases and cancer recurrence. They suggested that NK cells are critical mediators of metastasis formation immediately after surgery. Potential therapeutics with the capacity to target postoperative NK cell were also discussed. 

Some immune cells exhibit unexplained features and functions in our immune system. Natural killer T (NKT) cells possess the common features of T and NK cells in terms of their immune responses. In particular, invariant NKT (iNKT) cells, expressing an invariant TCR α-chain, constitute a major population of NKT cells [3]. In the review “The iNKT Cell-Macrophage Axis in Homeostasis and Disease”, Cruz et al. [4] described the role of NKT cells in homeostasis and disease. The authors discuss the details of iNKT cell activation upon macrophage engagement, as well as the modulation of macrophage functions and phenotypes after interaction with iNKT cells. Finally, the authors suggest that iNKT cell- and/or macrophage-based immunotherapeutic strategies may stand as promising targets for preventing disease onset and progression.

Five original articles introduce the novel findings of NK biology and immunotherapy under different contexts.

Cytokines are critical determinants of the phenotypic characteristics and functional activity of NK cells in immune response and development. In the article entitled “Pro- and Anti-Inflammatory Cytokines in the Context of NK Cell-Trophoblast Interactions”, Mikhailova et al. [5] introduced the effects of cytokines on NK cells in the presence of trophoblasts. Cytokines in the uteroplacental complex play important roles in the phenotype and functional state of NK cells during contact interaction with trophoblasts. The expression of critical receptors for NK effector function fluctuates with the cytokine treatment or co-culture. They demonstrated the effects of cytokines on NK cells in the presence of trophoblasts in an in vitro model.

In the article “NK Cell-Dependent Antibody-Mediated Immunotherapy Is Improved In Vitro and In Vivo When Combined with Agonists for Toll-like Receptor 2 in Head and Neck Cancer Models”, Gruijs et al. [6] investigated the effects of NK cell therapy combined with a Toll-like receptor (TLR) 2 agonist in a head and neck cancer model. The authors demonstrated that a cetuximab treatment, applied in combination with the immunostimulatory agonists of TLR 2, increased the release of several pro-inflammatory cytokines and chemokines by NK cells. The combination treatment of cetuximab and the TLR2 ligand Pam3CSK4 induced the infiltration of immune cells into tumors in comparison to mice that received cetuximab monotherapy and the results showed significant tumor regression. These findings suggest that the use of antibody-based immunotherapy with TLR stimulation is a promising treatment strategy for improving the clinical outcomes of cancer patients. 

In a short communication article entitled “A Chimeric IL-15/IL-15Rα Molecule Expressed on NFκB-Activated Dendritic Cells Supports Their Capability to Activate Natural Killer Cells”, Bosch et al. [7] reported that a chimeric IL-15/IL-15R molecule, expressed on NFκB-activated dendritic cells (DCs), activates NK cells. This group designed a chimeric protein consisting of IL-15 and IL-15 receptor (IL-15R). They showed that the fusion protein was detectable on the surface of DCs using mRNA electroporation, and increased the potential of NFκB-activated, IL-12-producing DC to activate NK cells. These results provide an initial proof-of-concept for improving cellular DC-based cancer immunotherapies.

In the article “Ex Vivo Expanded and Activated Natural Killer Cells Prolong the Overall Survival of Mice with Glioblastoma-like Cell-Derived Tumors”, Shida et al. [8] reported that highly purified and activated NK cells, designated as genuine induced NK cells (GiNKs), represent a promising immunotherapy for glioblastoma (GBM). The authors evaluated the antitumor effects of GiNKs in association with the programmed death 1(PD-1)/PD-ligand 1 (PD-L1) immune checkpoint pathway. The article claimed that GiNKs may represent a promising cell-based immunotherapy for patients with GBM and that they are minimally affected by the PD-1/PD-L1 immune evasion axis in GBM.

In the final article presented in this publication, “NLRP3 Deficiency in Hepatocellular Carcinoma Enhances Surveillance of NK-92 through a Modulation of MICA/B”, Lee et al. [9] demonstrated the crucial role of the NLRP3 inflammasome in hepatocellular carcinoma (HCC) via NK immunosurveillance. To examine the function of NLRP3 in HCC cells, the authors tested methods of cancer surveillance using NK cells in a co-culture of both cell types. NLRP3 knockout (KO) HCC cells showed the induction of MICA/B on the surface of HCC cells through the lowered expression of matrix metalloproteinases. A xenograft mouse model bearing NLRP3 KO HCC cells showed delayed tumor development and metastasis. These results suggest that the presence of NLRP3 KO in HCC enhances NK cell immunosurveillance via its interactionwith NKG2D-MICA.

As described in this Special Issue, NK cells are representative immune effector cells for the development of immunotherapies for hematological and solid cancer treatments. The possibility of using NK cells is growing rapidly, and will open up new opportunities and prospects for the development of immunotherapy in both hematological and solid cancer treatments; however, many questions remain to be elucidated in clinical trials and basic research. The results and discussions of these articles shed considerable light on NK biology and immunotherapy.
